# Systematic Review Reporting - Writing concisely and precisely

**DOI:** 10.12669/pjms.39.2.7428

**Published:** 2023

**Authors:** Patrick FW Chien, Khalid S Khan

**Affiliations:** 1 Patrick FW Chien, FRCOGm, Department of Obstetrics & Gynaecology, RCSI & UCD Malaysia Campus, 10450 Penang, Malaysia; 2 Khalid S Khan, FRCOG, Department of Preventive Medicine and Public Health, University of Granada, Faculty of Medicine, Granada, Spain. CIBER Epidemiology and Public Health, Madrid, Spain

**Keywords:** Systematic Review, Reporting, Integrity

## Abstract

Systematic reviews rank at the top of the evidence hierarchy. Concise writing implies drafting the systematic review article succinctly, i.e. using as few words to express as full an extent of the research effort as possible. Precise writing means drafting the text with accuracy especially with respect to the methodological and statistical aspects. The Abstract ought to be succinct and structured to allow for editors, peer reviewers and readers to get the gist of the key aspects of the systematic review with a quick read. The readership needs to be able to critically appraisal systematic reviews for their internal and external validity rapidly. The Abstract also needs to be standalone, representing an independent summary that can be fully understood without the need for reading the full paper. The standard structure of the main text of a scientific article called IMRaD (Introduction, Methods, Results and Discussion) applies equally to systematic reviews in the same way as it does to any other kinds of research manuscripts whether related to laboratory experiments or clinical trials. Restricting the word count limits to those imposed by journals may at first seem difficult, even unfair, to systematic reviewers. However, with the availability of online appendices to transparently and fully report the details of the methods, results and other aspects of the work undertaken allows for a succinct print or PDF article. Writing a shorter manuscript is more effortful than writing a longer report. This commentary is aimed at novice systematic reviewers to help them learn the written and unwritten writing rules in order to assist them in producing impactful publications to support evidence-based medicine.

## INTRODUCTION

Information generated from a well conducted systematic review provides the highest level of evidence to guide clinical practice.^1^ There has been an increased number of systematic reviews being published in biomedical journals in recent times (according to PubMed, over 40,000 last year).^2^,^3^ Hence, it is important that such manuscripts are written in an easily understandable style to allow the clinical effectiveness information to be disseminated effectively. Besides the use of clear and understandable language, manuscripts of systematic reviews also need to be written in a structured manner in the same way as an original research article is drafted for easier comprehension.^4^-^6^

Journal editors are keen for systematic review papers to be written as concisely and precisely as possible. Manuscripts of systematic reviews can be lengthy due to the enormous amount of information generated in this type of research. The tables summarising the included and excluded studies and the figures showing the study quality and forest plots of meta-analyses can be extensive, taking up many print pages of a journal. It is also important that relevant information pertaining to the design and conduct of a systematic review is reported in the manuscript in order for the internal validity of the findings to be accepted.

Hence, the challenge here is for these articles to provide the relevant information documenting accurately with as little print space as possible. Concise writing implies drafting the systematic review article succinctly, i.e. using few words to express the full extent of the research effort. Precise writing means drafting the text with accuracy especially with respect to the methodological and statistical aspects. For concise and precise writing, scientific terms should be used with specific meaning as shown in Table-I. For example, the term ‘meta-analysis’, which is merely a statistical analysis, should not be conflated with the term ‘systematic review’, which may or may not include meta-analysis. Lack of this precision in drafting text makes for confusion and misinterpretation.^7^

**Table-I:** Understanding basic research terminology deployed in writing systematic reviews precisely.

Term	Precise meaning
Systematic reviews	Research that summarizes the evidence on a clearly formulated question using transparent methods to identify, select and appraise relevant studies, and to extract, collate and report their findings.
Meta-analysis	A statistical technique for combining (pooling) the results of a number of studies addressing the same question to produce a summary result. A quality systematic review does not have to include a meta-analysis.
Transparency	Openness in reporting reviews clearly, accurately, honestly and completely. It includes many topics, e.g. reporting sources of funding and conflicts of interests.
Internal validity	The degree to which the results of a study or systematic review are likely to approximate the ‘truth’ for the research participants, i.e. are the results free of bias? It refers to the quality of the research and is a prerequisite for its external validity.
External validity	Also called generalizability or applicability, it is the extent to which the results observed in a study or a systematic review can be expected to apply in routine clinical practice, i.e. to people who did not directly participate in the research.
Evidence synthesis	A systematic approach to collating relevant evidence to address a research question. In addition to systematic reviews and meta-analyses there are umbrella reviews, network meta-analyses, guidelines, etc. within evidence syntheses.
Core outcomes	The minimum set of critical and important outcomes on which there is consensus among patients and practitioners that they directly measure what is clinically relevant.
Critical appraisal	Evaluation of systematic reviews for their internal and external validity.

Terms and precise meanings taken from: Khan KS, Zamora J. Systematic Reviews to support Evidence-Based Medicine. How to appraise conduct and publish reviews. 3rd Edition. (ISBN 9781032114675) London: Taylor & Francis Publishing 2022.

At the present moment, some of the systematic review articles are too long, with important information omitted or irrelevant data reported or even inaccurate titles.^7^ There may also be a lack of universally agreed structure in the reporting of such articles, as there are misperceptions about the originality of systematic reviews.^8^ The journals also differ in their word count limitations, e.g. the Cochrane Library reviews can be lengthy but the traditional journal article does not permit such excessive length. It is common for young researchers to undertake the systematic reviews as part of their postgraduate theses and these are also often reported in a peer review journal.

Such articles are sometimes submitted to journals in a version similar to that in the thesis chapter without taking into consideration that the readership for the version published in a biomedical journal is different to that for a postgraduate thesis. Writing a shorter article takes more effort than writing a longer manuscript, and it takes longer. The additional effort made by systematic review authors in drafting their manuscripts concisely and precisely is going to be appreciated by editors, peer reviewers and readers alike.

The purpose of this commentary is to provide some advice and guidance to novice researchers on the reporting of the findings of a systematic review for publication in a biomedical journal. Hopefully, new systematic reviewers can gain awareness of the written and unwritten publication rules we provide here in order to make it easier for their manuscripts to be accepted for publication on the first submission, reducing the time wasted in the rejection-resubmission cycle.

### Converting a thesis chapter into a journal article the initial steps:

The initial step is to adhere to the instructions to authors of the journal where the article is to be submitted. Different biomedical journals have different manuscript formatting requirements and word count limits on the manuscript length. There may also be limitations on the number of tables and figures which are permitted to be used in the print manuscript, these days produced as a PDF file. In this manner, publishers wish to ensure that the relevant information is provided at the minimum cost related to printing and electronic production. They are quite happy for the information that is not available in the printed manuscript to be provided as supplementary materials in appendices.

### Prospective registration:

Ideally, all systematic reviews should be prospectively registered in order to avoid unnecessary duplication of the same work by different research groups.^6^,^9^ Such registration is also encouraged to allow verification that the original systematic review protocol has been followed and any subsequent deviation from this protocol are fully reported with justifications in the published manuscript for the purpose of transparency.^10^ Hence, details of the registration should also be provided in the manuscript. There are many registration platforms e.g. Prospero (https://www.crd.york.ac.uk/prospero/) and OSF (https://accounts.osf.io/).

### Avoiding plagiarism:

There is also a need to avoid plagiarism, i.e. presenting text as your own that is not an identical copy of previously published text. This is important given that the issues discussed in the systematic review manuscript may be similar to those in the included primary studies.^11^ You may be able to carry out an electronic plagiarism check to ensure that the similarity index is acceptably low before the manuscript is submitted for publication.^12^ Paraphrasing and using quotations with references to the original sources protect against allegation of plagiarism. Most journals would carry out automated plagiarism checks, using artificial intelligence software programmes such as CrossCheck or iThenticate, and reject manuscript without peer review if large chunks of text are copied from other sources.

### Reporting checklists:

Journals also require submission of reporting checklists describing how the manuscript has been complied with guidance on transparent reporting. There are many checklists used to report systematic reviews and quality assessment tools, e.g. PRISMA^6^, MOOSE^13^, PRISMA-P^14^, GRIPP-2^15^, PRIOR^16^, AMSTAR^17^, ROBIS^18^, etc. We have provided a summary of these in Table-II. These checklists are intended to improve the quality of reporting of systematic reviews and peer reviewers often refer to these resources when assessing your papers.

**Table-II:** Checklists for transparent reporting, appraisal and concise of systematic reviews.

Name	Description of checklist
PRISMA^6^	Reporting of systematic reviews
MOOSE^13^	Reporting of meta-analyses of observational studies
PRISMA-P^14^	Reporting of systematic review and meta-analysis protocol
GRIPP-2^15^	Reporting of patient and public involvement in reviews
PRIOR^16^	Reporting for overviews of reviews or umbrella reviews
AMSTAR^17^	Reporting and quality appraisal tool for systematic reviews
ROBIS^18^	Tool to assess risk of bias in systematic reviews

### The Abstract of a systematic review:

The most important section of the manuscript is the Abstract as editors and peer reviewers as well as most general readership tend to scrutinise this part of the manuscript in the first instance. As the adage goes ‘a good first impression makes a lasting impression’. The Abstract is such an important element of the manuscript that we would advise not to leave writing the Abstract to the last moment before submission; we advise you to write it first and keep on revising it as you write the main text of your manuscript. Nowadays, it is common to employ a structured abstract with different subheadings on the Objective, Methods, Results and Conclusion.^6^

This is complementary to the structure of the main text and allows for the initially drafted Abstract to serve as a building block for the writing of the main text. Only the main findings and conclusion should be reported in the Abstract in order to the core message to be understood easily and quickly. Prospective registration details should also be provided in the Abstract. Beware that the Abstract needs to be able to standalone, being fully interpretable without the need for referring to the main article text.

### Structured manuscript of a systematic review:

Generally, the main text for such manuscripts should be written with the same structure as that used for original articles, i.e. Introduction, Methods, Results and Discussion, or IMRaD for short.^19^ You can include subheadings in the Methods, Results and Discussion sections in order to write systematically, avoiding duplications (Table-III).

**Table-III:** A systematic review manuscript structure.

Manuscript Structure (Suggested Length)	Comments (Written and unwritten rules)
Title (12-15 words)	Insert the term systematic review and meta-analysis or meta-regression or another term that describes other key features.
Authors	Give your identity as an author (registered, e.g. with ORCID; http://orcid.org/). Comply with International Committee of Medical Journal Editors authorship criteria (ICMJE; http://www.icmje.org). Single author systematic reviews are frowned upon as the extent of effort required frequently deploys at least double checking in the various steps of a systematic review.
Abstract (250 words or more if permitted by the journal)	Write a structured Abstract with at least four sub-headings, Objective, Methods, Results, and Conclusion, matching the main text structure. Some journals may require additional sub-headings.
Main manuscript (3000-5000 words)	IMRaD structure or Introduction, Methods, Results and Discussion. Follow the journal’s instructions.
Introduction (350 words; 3 paragraphs)	Give disease prevalence, effect in life quality, economic impact, etc. (first paragraph), and justification of your systematic review in light of deficiencies of previous studies applying AMSTAR-2 or ROBIS (second paragraph), and repeat the objective drafted in the Abstract (third paragraph).
Methods, Results (750 words each)	Prepare in line with a reporting guideline like MOOSE. Give registration details. The Methods and Results sub-sections should be complementary. We advise 3 sub-sections each under sub-headings covering study search and selection, data extraction and study quality assessment, and data synthesis. Tables and figures are usually provided at the end of the manuscript and are incorporated in the main text following acceptance of the manuscript.
Discussion (1000 words; 4-5 paragraph)	Give main findings, strengths and limitations, interpretation of findings, their implications for practice and research, before drawing the conclusion. Write these with sub-headings first and if your chosen journal does not permit them, just take them out before submission.
Acknowledgements	Those who contributed to the review, but not sufficiently to meet authorship criteria, should be named here.
Disclosure of interest	A formal disclosure may be mandatory using an ICMJE form.
Bibliography	Ensure compliance with authors’ instruction. If there are limits to permitted number so references, provide the references to included and excluded studies in appendices.
Appendices	Give detail of searches, data used to construct figures, etc. Full descriptions of the abbreviations used would need to be repeated even when provided in the Abstract and the main text, using footnotes if required.

Adpated from: Khan KS, Zamora J. Systematic Reviews to support Evidence-Based Medicine. How to appraise conduct and publish reviews. 3rd Edition. (ISBN 9781032114675) London: Taylor & Francis Publishing 2022.

The Introduction should describe the clinical problem being addressed in the article and its importance. A paragraph to justify the rationale for undertaking the systematic review should be presented here. If a similar review has been published previously, the Introduction should explain the need for an update, e.g. there may have been new, large studies published since the last review. There may be many reviews published on the topic, so inevitably previous reviews would need to be critically appraised. The Introduction doesn’t really permit a detailed appraisal description, so an appendix tabulating the critique of the previous reviews using e.g. AMSTAR-2 or ROBIS tools can be supplemented.

To end the Introduction section, the authors should explicitly state the research question based on a structured format with the health outcomes defined a priori. Core outcomes, the minimum set of critical and important outcomes on which there is consensus regarding clinical relevance among patients and practitioners^20^, offer a focus for the main article, leaving non-core outcome data for reporting in appendices. Editors use the information provided in the Introduction to determine if the submission should be given priority over other manuscripts they have in front of them.

The Methods should start by giving the prospective registration details and the reporting checklists used. The structured question forms the basis of the search strategy. The search term combination and the databases searched with dates need to be reported in a reproducible manner.^21^ The inclusion and exclusion criteria for articles to be used in or omitted from the systematic review should be also reported.^6^ The instrument used for the assessment of study quality and the method of data extraction should be reported as well as the assessment on how any disagreement between the systematic reviewers was resolved.^6^

The level of agreement between the systematic reviewers should also be assessed and reported. The statistical method for pooling data, subgroup analysis, sensitivity analysis and assessment of publication bias also have to be reported in the manuscript.^6^ If there has been public involvement^24^ in the review, this should also be reported in the Methods. Importantly, if there have been modifications to the systematic review since its registration, e.g. if the nature changed to scoping review in light of the emerging findings during the review work, these should be justified in the Methods section openly.

The descriptions of the literature searches, the lists of included and excluded articles along with the reasons for such decisions, the details of data extraction methods and study quality assessment can take up too much space. This is where supplementary appendices giving the search strategy, excluded studies with reasons, the data extraction and quality assessment checklist help with completeness of reporting. Peer reviewers and readers can further scrutinise these details if they wish.

The Results section should incorporate a flow chart on the search and subsequent inclusion and exclusion of articles into the systematic review with a brief description of the included articles. The information on the included studies allows the peer reviewers and readers to determine the comprehensiveness of the literature search and also the level of external validity of the findings from the review. The findings of the study quality assessment should be then described in order for the peer reviewers and readers to determine the robustness of the evidence from the review. These can be succinctly presented in a figure using 100% stacked bar chart, providing the actual number of studies with particular features within the bars (easily constructed in a spreadsheet).

To conserve space, tables of individual study characteristics and quality can be provided in appendices. The main findings on the primary outcome(s) should be reported followed by the findings based on the secondary outcomes. Forrest plots should be provided for at least the primary outcome(s) together with assessment on possible heterogeneity in the pooled data. Results of any sensitivity or subgroup analyses together with the assessment of possible publication bias also should be briefly reported.^6^

Again, all the outputs from such analyses should be provided as appendices so that they can be scrutinised by those who wish to ensure integrity of the review. Appendices, tables and figures should have titles with sufficient details to permit them to standalone and assist in understanding their contents without the need to refer to the Abstract or the main text. Any additional results tables and figures which may not be permitted to be published in the PDF version of the manuscript should be available as supplementary material. In this way, the systematic review can be fully presented without taking up an unacceptably high amount print space for the accepted article.

The Discussion section should generally consist of no more than 4-5 paragraphs. The main findings of the systematic review and any clinical implications to clinical practice should be summarised in the first paragraph (without repeating the numerical data presented in the Results section). This is followed by a discussion of the strengths and limitations of the systematic review in the next paragraphs. The subsequent paragraph should address the interpretation of the findings in relation to other previously published reviews on the same topic. Any interpretation of findings may cover the issue of cost effectiveness briefly even though this may not be the main focus of the systematic review.

The implications for clinical practice and future research should then be described here as well. The last paragraph should provide the conclusion of the systematic review based only on the findings concerning the primary outcome(s) determined a priori as stated in the Introduction section. Authors should avoid overstating the conclusion of the review, especially e.g. in situations when data from observational studies are pooled together with randomised trials and there is significant heterogeneity in the results.

### Integrity and transparency:

The roles and contributions of the authors^22^, all potential conflicts of interest^6^, acknowledgement(s), sources of funding^23^, etc. should also be reported to meet the openness agenda that is critical to public trust in science. The interest of transparency in reporting is served by providing the above in full detail using the International Committee of Medical Journal Editors criteria and forms (ICMJE; http://www.icmje.org), supplied as appendices if required. Reporting checklists should be provided as an appendix to explicitly demonstrate how and where the article complies, or justifiably not comply, with the requirements for transparent reporting.

### Dealing with peer review comments:

In dealing with peer review comments, it is important that responses should be submitted within the time period given by the editors dealing with the submission. If this is not possible for any reason, authors should contact the journal office to request for time extension. The request should be justified. Frequently the need to respond to peer review may require update searches and the editors will understand and accept if this was the reason for the extension requested. Each comment will need to be itemised with an appropriate response and any change in the manuscript highlighted in order for the editor to be able to be easily assess and track the changes made to the manuscript. Editors may request the original peer reviewers to address the responses you provide.

It is important to candidly acknowledge any limitations of the systematic review highlighted by the peer reviewers and to include (where possible) any additional information into the manuscript. If there are too many comments, as is the case when there are many peer reviewers, tabulation of responses is most helpful in demonstrating that that critique received has been addressed in a structured and comprehensive manner.^25^ Beware that the response to editors’ and peer reviewers’ comments need to be detailed in order for it to be convincing. The arguments made may have to be backed by references and scientific explanations.

There may be difference of opinion over how particular issues should be handled, e.g. heterogeneity is not always easily explained and there may be unexplained heterogeneity that is unavoidable. It may require you to write quite a long table of responses. The response document may in fact be longer than the manuscript itself in some circumstances. Authors need to be concise and precise in responding too but there are not rigid word limits applied in the same way as those applied to manuscripts. In the future it is likely the peer review will be openly published to maximise transparency, and going forward responses to peer review comments and the original and revised manuscripts will all be publicly available.^26^

## CONCLUSION

Writing concisely and precisely is effortful and time-consuming. Editors, peer reviewers and readers need to be able to critically appraisal systematic reviews for their internal and external validity rapidly. It is important that systematic reviews, even when written within word count limits, can be fully understood so that the data from such work can be used to shape clinical practice and policy. A structured Abstract, mimicking the main text structure, is the key to writing convincingly. The standard structure of the main text of a paper, abbreviated in the acronym IMRaD, applies equally to systematic reviews as it does to any other kinds of research types including laboratory experiments and clinical trials. By reporting the systematic review findings in a succinct manuscript, accompanied with details transparently and completely presented in supplementary files, the data collated can be accurately presented to ensure that critical appraisal and assimilation of evidence into practice is facilitated.

### Authors’ Contribution:

**PFWC:** Wrote, edited and approved the manuscript.

**KSK:** Edited and approved the manuscript.
